# Genetically engineered nanomodulators elicit potent immunity against cancer stem cells by checkpoint blockade and hypoxia relief

**DOI:** 10.1016/j.bioactmat.2024.04.008

**Published:** 2024-04-23

**Authors:** Yuanwei Pan, Ling Yu, Lujie Liu, Jing Zhang, Shuang Liang, Badri Parshad, Jialin Lai, Li-Min Ma, Zhaohui Wang, Lang Rao

**Affiliations:** a The Research and Application Center of Precision Medicine, The Second Affiliated Hospital of Zhengzhou University, Zhengzhou, 450014, China; b Institute of Biomedical Health Technology and Engineering, Shenzhen Bay Laboratory, Shenzhen, 518132, China; c State Key Laboratory of Bioactive Substance and Function of Natural Medicines, Institute of Materia Medica, Chinese Academy of Medical Sciences & Peking Union Medical College, Beijing, 100050, China; d Department of Critical Care Medicine, Guangdong Provincial Hospital of Chinese Medicine, Guangzhou, 510120, China; e Medical Research Center, Guangdong Provincial People's Hospital (Guangdong Academy of Medical Sciences), Southern Medical University, Guangzhou, 510080, China; f Wellman Center for Photomedicine, Massachusetts General Hospital, Harvard Medical School, Boston, MA, 02129, USA

**Keywords:** Cancer immunotherapy, Cancer stem cells, Hypoxia, CD47-SIRPα, Cell membrane vesicles

## Abstract

Rapid development of checkpoint inhibitors has provided significant breakthroughs for cancer stem cell (CSC) therapy, while the therapeutic efficacy is restricted by hypoxia-mediated tumor immune evasion, especially hypoxia-induced CD47 overexpression in CSCs. Herein, we developed a genetically engineered CSC membrane-coated hollow manganese dioxide (hMnO_2_@gCMs) to elicit robust antitumor immunity by blocking CD47 and alleviating hypoxia to ultimately achieve the eradication of CSCs. The hMnO_2_ core effectively alleviated tumor hypoxia by inducing decomposition of tumor endogenous H_2_O_2_, thus suppressing the CSCs and reducing the expression of CD47. Cooperating with hypoxia relief-induced downregulation of CD47, the overexpressed SIRPα on gCM shell efficiently blocked the CD47-SIRPα “don't eat me” pathway, synergistically eliciting robust antitumor-mediated immune responses. In a B16F10-CSC bearing melanoma mouse model, the hMnO_2_@gCMs showed an enhanced therapeutic effect in eradicating CSCs and inhibiting tumor growth. Our work presents a simple, safe, and robust platform for CSC eradication and cancer immunotherapy.

## Highlights

• Hypoxia relief by hMnO_2_@gCMs could suppress CSCs and reduce CD47 expression.
• hMnO_2_@gCMs block the CD47-SIRPα pathway *via* overexpressed SIRPα on gCMs shell.
• Hypoxia relief-induced downregulation of CD47 and CD47-SIRPα blockade synergistically elicit robust antitumor immune responses.
• hMnO_2_@gCMs present enhanced therapeutic efficacy in eradicating CSCs.

## Graphical abstract

A novel genetically engineered biomimetic nanoplatform was developed to trigger the immunity against the CSCs through hypoxia relief and synergetic inhibition of the CD47-SIRPα signaling pathway. The work presented here provides a simple, safe, and effective strategy in activating body's immune responses for eradication of the CSCs.

**Figure:**
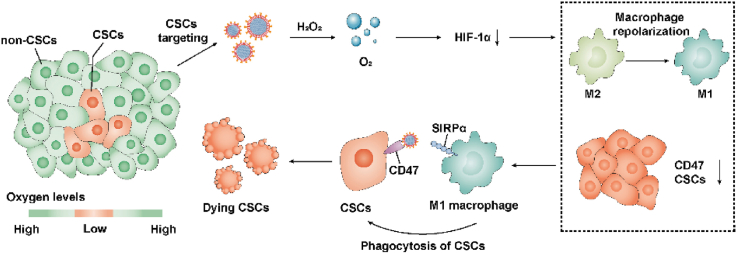

## 1 Introduction

Cancer stem cells (CSCs) are a small subpopulation with self-renewal, high tumorigenicity, multi-differentiation potential and therapeutic resistance, and are related to tumor progression, recurrence, and metastasis [[1], [2], [3]]. Conventional cancer treatments are not effective in killing CSCs, moreover, CSCs exhibit elevated DNA damage repair ability, quiescence, efflux pumps, and high resistance to apoptosis, making the eradication of CSCs more difficult [[4], [5], [6], [7]]. The immune checkpoint blockade (ICB) therapy can yield a durable immune response in several malignancies and has attracted tremendous attention in suppressing CSCs [[8], [9], [10]]. Recent studies suggested that immune checkpoints such as programmed cell death 1-ligand 1 (PD-L1), CD276, and CD47 are upregulated in CSCs, which helps them evade immune surveillance in tumor initiation, progression, and metastasis [[11], [12], [13], [14]]. Especially, CD47, a ligand for signal regulatory protein α (SIRPα) expressed on macrophages to block phagocytosis, has been identified as an important mechanism for CSCs to evade macrophage phagocytosis through the CD47-SIRPα “don't eat me” pathway, which is also important for the maintenance of CSCs [[15], [16], [17]]. Therapeutic antibodies against CD47 have shown a striking anti-CSCs activity, while immune evasion limits its clinical outcomes [18].

Tumor hypoxia, a typical feature of solid tumors, is caused by insufficient blood supply and abnormal vascular structure of the tumor [19,20]. Tumor hypoxia is often associated with immune evasion, which limits the effects of immunotherapy against CSCs [21,22]. Various studies have corroborated that CSCs reside in hypoxic tumor regions, thereby favoring their survival, stemness maintenance, and evolution [[23], [24], [25]]. Under a hypoxic microenvironment, the CSCs exhibit a poorly differentiated state to maintain their self-renewal, and the CSCs increase in a process mediated by hypoxia-inducible factor-1α (HIF-1α) [26]. In addition, recent studies have revealed that the hypoxic tumor microenvironment could tune tumor-associated macrophages (TAM) into the tumorigenic M2-like phenotype [27]. More importantly, it has been reported that HIF-1α can directly activate the transcription of the CD47 gene in CSCs, thereby evading macrophage phagocytosis [16]. To date, although CD47 inhibition has become an important breakthrough in suppressing CSCs, challenges still exist because the invisible status of hypoxic CSCs enables them to evade immune surveillance [28,29]. Consequently, synergetic inhibition of the CD47-SIRPα signaling pathway by downregulating CD47 expression through hypoxia alleviation and CD47-SIRPα blockade to elicit an immune response and enhance anti-CSC efficacy would be critically important.

The CSCs are located in the central region of tumors [30], thus identifying novel potent targeting strategies that can break through various physiological barriers and boost the accessibility of therapeutic agents to hypoxic CSCs is key to improving the efficiency of tumor treatment [31,32]. At present, various attempts have been devoted to target hypoxic CSCs, including delivering hypoxia-activated prodrug to hypoxic CSCs and using hyperbaric oxygen to disrupt hypoxia [25]. However, limited CSC targeting and short oxygen duration are insufficient to achieve satisfactory therapeutic outcomes [28]. Recently, cancer cell membrane-coated nanoparticles have been considered as a feasible strategy to target tumors through homologous targeting ability, especially the CSC membrane has been proven to enhance the targeting effect on CSCs [33,34]. Moreover, through the genetic engineering approach, the engineered cell membrane can display specific immune checkpoint proteins, thereby enhancing cancer immunotherapy by disrupting the immune checkpoint signaling pathway [[35], [36], [37]]. Meanwhile, our previous research has proven that engineered cell membrane nanovesicles could effectively improve the therapeutic efficacy of cancer [38,39]. Considering the advantages offered by engineered cell membranes, we anticipate that they can enable efficient CSC targeting and CSC eradication [40].

**Fig. 1:**
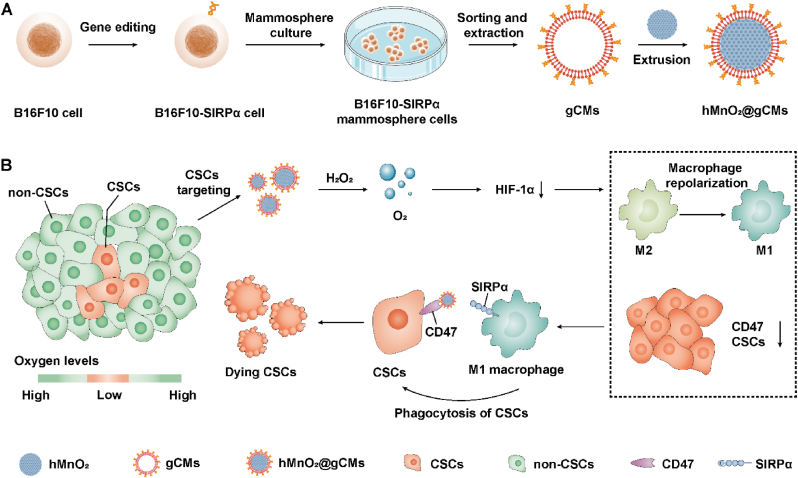
Schematic illustration of hMnO_2_@gCMs elicit potent antitumor immunity against CSCs. (A) hMnO_2_@gCMs nanoparticles were obtained by coating CSC membranes that overexpressing SIRPα variants on hMnO_2_ nanoparticles. (B) hMnO_2_@gCMs nanoparticles robust antitumor immune responses *via* CD47-SIRPα blockade and hypoxia relief.

In this study, we developed a genetically engineered CSC membrane-coated hollow manganese dioxide nanoparticle (hMnO_2_@gCMs) to relieve tumor hypoxia and enhance immune checkpoint blockade therapy against CSCs (Fig. 1A). The hMnO_2_ core could relieve tumor hypoxia by decomposing H_2_O_2_ to produce O_2_, with the relief of hypoxia, the CSCs niche would be destroyed and the CD47 expression would be downregulated, thereby enhancing the immune response. In addition, the gCMs shell could enhance the CSCs targeting and the genetically overexpressing SIRPα variants can efficiently block CD47-SIRPα signaling pathway and strengthen immunotherapy to eradicate the CSCs (Fig. 1B). This study demonstrated that as-prepared biomimetic hMnO_2_@gCMs nanoplatforms have excellent therapeutic efficacy and manifest great potential in the eradication of CSCs.

## 2 Materials and methods

### 2.1 Reagents

Triton X-100, cyclohexane and n-Hexanol were purchased from Alfa Aesar. Tetraethyl orthosilicate (TEOS) and (3-Aminopropyl) triethoxysilane (APTES) were obtained from Merck. Potassium permanganate (KMnO_4_), sodium carbonate (Na_2_CO_3_) and 30 wt% hydrogen peroxide (H_2_O_2_) were brought from Sinopharm Chemical Reagent Co., Ltd.

### 2.2 Synthesis of hMnO_2_ nanoparticles

Firstly, 5.3 mL of Triton X-100, 22.5 mL of cyclohexane, 0.75 mL of ammonia, 5.4 mL of n-Hexanol and 1 mL of H_2_O were mixed in a flask and stirred for 30 min. Then, 0.5 mL of TEOS and 0.1 mL of APTES were added to the mixed solution and stirred for 24 h. The obtained silica nanoparticles (SiO_2_) nanoparticles were collected and washed three times, then dispersed in ultrapure water (1 mg mL^−1^). 15 mL of KMnO_4_ (10 mg mL^−1^) was added dropwise into the 20 mL of SiO_2_ solution, and then continuous ultrasound for 6 h KMnO_4_ was reduced by organosilica present on the surface of SiO_2_ to obtain a uniform mesoporous MnO_2_ layer (MnO_2_@SiO_2_). The MnO_2_@SiO_2_ nanoparticles were collected and etched by Na_2_CO_3_ solution (2 M, 20 mL) to obtain the hollow mesoporous MnO_2_ nanoparticles (hMnO_2_).

### 2.3 Synthesis of hMnO_2_@gCMs nanocomposites

The preparation of SIRPα variant-engineered B16F10 cells has previously been reported by our group [38]. To obtain B16F10-SIRPα tumorsphere cells enriched with CSCs, the well-established suspension culture was used. The sorting and identification of CD133^+^ CSCs was performed using a BD Aria III. gCMs encapsulation was prepared according to our previous literature [38]. Briefly, B16F10-SIRPα CSCs were disrupted by ultrasonic homogenizer and then centrifuged (2000 g, 20 min) to remove organelles and other cell inclusions. The supernatants were further centrifuged (20,000 g, 30 min) and the pellet was discarded. After that, the supernatant was collected by an ultra-speed centrifuge (80,000 g, 2 h). The pellets were resuspended in PBS mixed with protease inhibitor tablet and extruded through 400 nm and 200 nm nanopore polycarbonate membranes using an Avanti mini-extruder. Subsequently, gCMs and hMnO_2_ were mixed and then orderly extruded through a 200 nm pores, the obtained hMnO_2_@gCMs were collected by centrifugation and maintained at 4 °C for future use.

### 2.4 Instruments

The morphology of nanoparticles was verified by TEM (Talos F200X). The dynamic light scattering (DLS) and zeta potential was carried out by Zetasizer Pro (Malvern Panalytical). The UV–vis spectra were characterized by UV–vis spectrophotometer (UV-2600i, Shimadzu Corporation). The Mn content was detected by ICP-OES (Optima 2100, PerkinElmer). XPS analysis was conducted by an Axis Ultra DLD instrument. The dissolved oxygen was performed on an oxygen probe (ST300 D, OHAUS).

### 2.5 Sodium dodecyl sulfate-polyacrylamide gel electrophoresis (SDS-PAGE) analysis

gCMs, hMnO_2_, and hMnO_2_@gCMs were resuspended with protein extraction buffer and heated at 95 °C for 5 min. The samples (20 μg each) were loaded into a 10 % SDS-polyacrylamide gel and run at 120 V for 2 h. After staining with Coomassie blue for 4 h, the gel was decolorated overnight before observation.

### 2.6 Cell culture

The B16F10 cell lines were cultured with Dulbecco's modified Eagle's medium (DMEM) that contained 10 % fetal bovine serum and 1 % penicillin/streptomycin and were cultivated under 37 °C within 5 % CO2. B16F10 tumorsphere cells were cultured with DMEM/F12 containing B27, 0.4 % bovine serum albumin, 20 ng mL^−1^ epidermal growth factor and 20 ng mL^−1^ fibroblast growth factor-basic in an ultralow attachment plate.

### 2.7 In vitro biocompatibility

B16F10 tumorsphere cells (1 × 10^4^ cells per well) were seeded in an ultralow attachment 96-well plate for 24 h, and then cultured with 20–100 μg mL^−1^ hMnO_2_, gCMs, hMnO_2_@gCMs for another 24 h. The cytotoxicity was evaluated using cell counting kit-8 (CCK-8) assay.

### 2.8 Cellular uptake assay

After seeded in confocal dish (2 × 10^4^ cells per well) for 24 h, B16F10 tumorsphere cells were incubated with hMnO_2_@gCMs for another 2, 4 or 8 h, the hMnO_2_@gCMs was stained with DiD in advance. After being stained with DAPI and phalloidin-FITC, the cells were observed under a confocal laser scanning microscopy (CLSM, ZEISS LSM980). To determine the cellular uptake by flow cytometry (FACS), the tumorsphere cells were incubated overnight and then incubated with hMnO_2_@gCMs labeled with DiO for 2, 4 or 8 h. After staining with PE anti-mouse CD133 (Biolegend, 141203), the cells were detected by FACS. For lysosomal escape analysis, 24 h after seeding in confocal dish (2 × 10^4^ cells per well) for, CSCs were incubated with DIO-labeled hMnO_2_@gCMs for additional 1, 2, 4 or 6 h. After being stained with Lysotracker Red, the cells were observed under CLSM.

### 2.9 Determination of HIF-1α expression at cellular level

To evaluate the proportion of HIF-1α after hypoxia relief, the tumorsphere cells (2 × 10^4^ cells per well) were cultured in confocal dish for 48 h under hypoxic conditions, and then incubated with hMnO_2_ or hMnO_2_@gCMs under hypoxic conditions for 24 h. The HIF-1α expression level of the cells with different treatment was detected by the immunofluorescence staining.

### 2.10 Identification of stemness-related property after hypoxia relief

The tumorsphere cells (1 × 10^5^ cells mL^−1^) were seeded onto ultralow attachment plate for 48 h under hypoxic conditions and then incubated with hMnO_2_ for another 24 h. Afterwards, the mRNA levels of Sox2, Nanog, and Oct4 were evaluated by Quantitative real-time polymerase chain reaction (qRT-PCR) based on our previously reported method [41].

### 2.11 Tumorspheres-forming assay

The tumorsphere cells (1 × 10^5^ cells mL^−1^) were seeded onto ultralow attachment plate for 48 h under hypoxic conditions and then incubated with hMnO_2_ or hMnO_2_@gCMs for another 24 h. Afterwards, the treated cells were dissociated and seeded at 2000 or 4000 cells per well. The number of newly formed tumorspheres (diameter >50 μm) was recorded under microscope after 10 days of incubation.

### 2.12 Determination of CSCs proportion after *in vitro* hypoxia relief

The tumorsphere cells were treated with hMnO_2_ or hMnO_2_@gCMs as described above. After that, the tumorsphere cells were stained with PE anti-mouse CD133, and the proportion of CD133^+^ cells were analyzed by FACS.

### 2.13 Animals and *in vivo* biocompatibility

All the animal experiments were performed in accordance with the guidelines approved by the animal research ethics committee at Shenzhen Bay Laboratory (Permit No. AERL202203). To evaluate the *in vivo* biocompatibility of the nanoparticles, C57BL/6 mice were injected with PBS or hMnO_2_@gCMs (100 μL, 20 mg kg^−1^) *via* the tail vein. After 4 weeks, blood samples were extracted from the mice for complete blood count and blood chemistry tests. Major organs from various group were harvested for hematoxylin and eosin (H&E) staining.

### 2.14 In vivo CSC tumor model

The B16F10–CSCs bearing mice melanoma model was established by subcutaneous injection of B16F10–CSCs tumorsphere cells (2.5 × 10^5^ cells for one mouse) in the back of 4 to 6-week-old female C57BL/6 mice. The tumor volume was measured by a caliper and estimated by the equation: tumor volume = L × W^2^/2, where L, W are the tumor length and tumor width, respectively.

### 2.15 Determination of hypoxia relief *in vivo*

When the subcutaneous tumor volume reached about 50–100 mm^3^, 100 μL of PBS, hMnO_2_, or hMnO_2_@gCMs (dose of hMnO_2_ = 10 mg kg^−1^, gCMs = 10 mg kg^−1^) were injected *i.v.* into B16F10–CSCs tumor-bearing mice. After 24 h, the mice were sacrificed and tumor were harvested. The expression levels of HIF-1α, CD47 and CD133 in tumor tissues were detected by immunofluorescence staining.

### 2.16 In vivo bio-distribution

100 μL of hMnO_2_ or hMnO_2_@gCMs at a dose of 10 mg kg^−1^ based on hMnO_2_ were injected *i.v.* into B16F10–CSCs tumor-bearing mice. After 24 h, the major organs and tumors of mice were harvested and weighed, then the Mn content was quantified by ICP-OES. For *in vivo* fluorescence imaging, the hMnO_2_ and hMnO_2_@gCMs was first labeled with IR780. After *i.v.* injection of IR780-labeled hMnO_2_ and hMnO_2_@gCMs into B16F10–CSCs tumor-bearing mice, the mice were tracked by IVIS Spectrum imaging system (IVIS® Lumina III PerkinElmer). To evaluate targeted ability to CSCs *in vivo*, the hMnO_2_ and hMnO_2_@gCMs was first labeled with DID. And then tumor-bearing mice received an *i.v.* injection of DID-labeled hMnO_2_ and hMnO_2_@gCMs. Furthermore, the tumors were stained with CSC marker CD133 and DAPI at 24 h post-injection.

### 2.17 In vivo antitumor study

B16F10–CSCs tumor-bearing mice were randomly assigned to four groups and treated with PBS, hMnO_2_, gCMs, or hMnO_2_@gCMs (dose of hMnO_2_ = 10 mg kg^−1^, gCMs = 10 mg kg^−1^) at every other day for three times. The tumor volume and body weight were measured every two days, and two weeks after the first treatment, the tumors were stained with H&E and Ki-67, and the main organs were dissected for H&E staining.

To evaluate the efficacy of chemo-immunotherapy combination therapy, B16F10–CSCs tumor-bearing mice were randomly assigned to four groups and treated with PBS, DOX, hMnO_2_@gCMs, or hMnO_2_@gCMs (DOX) (dose of hMnO_2_ = 10 mg kg^−1^, gCMs = 10 mg kg^−1^, DOX = 5 mg kg^−1^) at every other day for three times. The tumor volume and body weight were recorded every two days for two weeks.

### 2.18 Expression of CSCs markers *in vivo*

B16F10–CSCs tumor-bearing mice were treated with PBS, hMnO_2_, gCMs, or hMnO_2_@gCMs (dose of hMnO_2_ = 10 mg kg^−1^, gCMs = 10 mg kg^−1^) at every other day for three times. Two weeks after the first treatment, the tumor slices were stained with CSCs markers CD133, Sox2, Nanog.

### 2.19 Flow cytometry

Tumor tissues were first harvested after the therapy and digested with culture medium supplemented with digestive enzyme, and then filtered through 70 μm cell strainers to obtain single cell suspensions. For macrophage polarization, cells were stained with anti-CD45-Cy5.5, anti-F4/80-AlexaFluor647, anti-CD11b-FITC, anti-CD80-Bv510 and anti-CD206-Bv785 antibodies for 30 min. For T cells detection, the cells were stained with anti-CD45-Cy5.5, anti-CD3-Bv510, anti-CD4-Bv650 and anti-CD8-AlexaFluor750 antibodies for 30 min. The samples were washed with PBS and then measured by flow cytometer (Beckman).

### 2.20 Immunofluorescence staining assay

Tumor tissues were harvested, fixed and embedded in paraffin. For characterization of immune cells infiltration, tumor histologic slices were immunofluorescence stained with primary antibodies anti-CD8 rabbit mAb, anti-F4/80 rabbit mAb and secondary antibodies Alexa Fluor 488 of goat anti-rabbit IgG, Alexa Fluor 647 of goat anti-rabbit IgG. After staining with DAPI, the slices were imaged under CLSM.

### 2.21 Cytokine detection

B16F10–CSCs tumor-bearing mice were randomly assigned to four groups, PBS, hMnO_2_, gCMs, or and hMnO_2_@gCMs. Mice were received three doses of different nanoparticles (dose of hMnO_2_ = 10 mg kg^−1^, gCMs = 10 mg kg^−1^) by *i.v.* injection every other day. Two weeks after the first treatment, tumor tissues of different groups were obtained. The intratumor levels of tumor necrosis factor alpha (TNF-α), and interferon gamma (IFN-γ) were measured by using corresponding ELISA kits under the guidelines provided by manufacturers.

### 2.22 Statistical analysis

All data are expressed as mean ± standard error of the mean (S.E.M.) with independent experiments. The number of samples (n) in each group is specified in the figure legend. Statistical analysis was carried out using Origin 9.0 software. Comparison of parameters for two group were performed by the unpaired two-tailed *t*-test. Comparison of parameters for multiple groups were performed by one-way analysis of variance (ANOVA) with the Tukey significant difference post hoc test. *P* values less than 0.05 were considered statistically significant, and significance levels were set at (*) for *P* < 0.05, (**) for *P* < 0.01, and (***) for *P* < 0.001.

## 3 Results and discussion

### 3.1 Preparation and characterization of hMnO_2_@gCMs nanoparticles

**Fig. 2:**
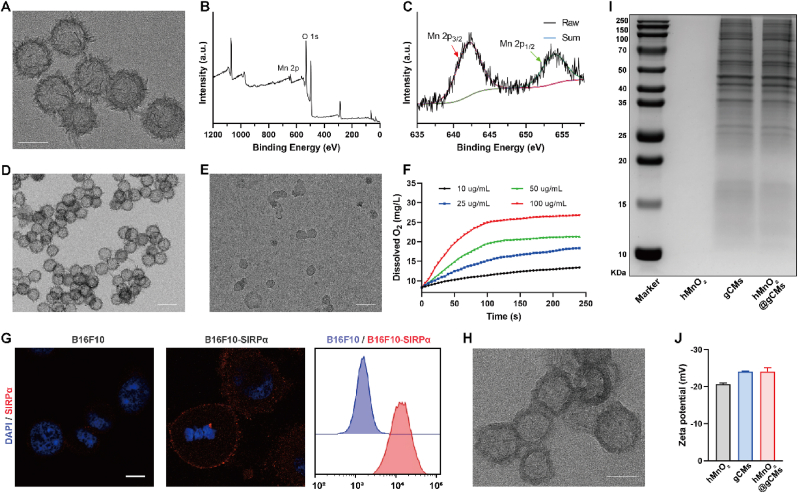
Preparation and characterizations of hMnO_2_@gCMs. (A) TEM images of hMnO_2_ NPs. Scale bar, 50 nm. (B) XPS full survey spectra and (C) High resolution of Mn 2p spectrum of hMnO_2_ NPs. TEM images of hMnO_2_ NPs after incubation in (D) PBS (pH 7.4) and (E) PBS (pH 5.5) containing H_2_O_2_ (100 μM). Scale bar, 100 nm. (F) Changes in O_2_ concentration in H_2_O_2_ solutions (100 μM) after adding different concentrations of hMnO_2_. (G) Confocal images and FACS analysis of SIRPα expression level on B16F10 and B16F10- SIRPα cells. Scale bar, 10 μm. (H) TEM images of hMnO_2_@gCMs NPs. Scale bar, 50 nm. (I) SDS-PAGE analysis of hMnO_2_, gCMs and hMnO_2_@gCMs. (J) Zeta potential of hMnO_2_, gCMs and hMnO_2_@gCMs NPs.

The synthesis process of hMnO_2_ is shown in Fig. S1 [42]. As revealed by the TEM images (Fig. 2A), the hMnO_2_ displayed spherical morphology and hollow structure. The XPS spectra of hMnO_2_ showed the signals of O and Mn elements (Fig. 2B and Fig. S2), the characteristic peaks at 642.4 and 654.2 eV in the Mn 2p spectrum could be attributed to the Mn 2p_3/2_ and Mn 2p_1/2_, respectively (Fig. 2C). The hMnO_2_ could catalyze the decomposition of H_2_O_2_ into water and oxygen and simultaneously degrades itself into water-soluble Mn^2+^ ions, thereby reducing the long-term toxicity of hMnO_2_ [27]. The morphology of hMnO_2_ degraded by H_2_O_2_ was examined by TEM analysis. The hMnO_2_ showed negligible change in pH 7.4 solution (Fig. 2D); however, in the presence of acidic H_2_O_2_ solution, hMnO_2_ was significantly degraded (Fig. 2E). The degradation rates were further determined by the change in the hMnO_2_ characteristic absorbance band, which was stable at pH 7.4 but decreased rapidly in the presence of an acidic H_2_O_2_ solution (Fig. S3A). We found that hMnO_2_ could rapidly trigger O_2_ generation in a concentration-dependent manner (Fig. 2F). The O_2_ bubbles generated by hMnO_2_ were visible to the naked eye (Fig. S3B).

To construct the genetically engineered CSC membranes (gCMs), SIRPα variant was first transduced onto B16F10 murine melanoma by lentivirus [38,39]. To obtain B16F10-SIRPα tumorsphere cells enriched with CSCs, the well-established suspension culture was carried out (Fig. S4) [28]. The immunofluorescence imaging and flow cytometry clearly revealed the expression of SIRPα variants on CSCs (Fig. 2G). Afterwards, the CSC membranes were prepared by hypotonic lysing and then coated on the surface of hMnO_2_ to produce hMnO_2_@gCMs. The TEM images demonstrated that the hMnO_2_@gCMs were coated by a layer of the gCMs membrane (Fig. 2H). SDS-PAGE assay was further performed to verify the successful coating of gCMs. As shown in Fig. 2I, the proteins of gCMs membranes were extensively retained on the hMnO_2_@gCMs. The surface charge of the hMnO_2_@gCMs was found to increase roughly to the level of gCMs after the coating as verified by zeta potential measurement (Fig. 2J). Furthermore, DLS analysis showed that the hydrated particle size distribution of hMnO_2_@gCMs was uniform, with negligible size changes observed over 10 days (Fig. S5). The degradation rate of hMnO_2_@gCM at different pH was further determined by the change of the characteristic absorption band, which was stable at pH 7.4, but decreases rapidly under acidic conditions. Considering the acidic environment of tumor tissue, the coating of gCM will not affect the degradation of hMnO_2_ (Fig. S6).

### 3.2 Biocompatibility and CSCs targeting ability of hMnO_2_@gCMs

**Fig. 3:**
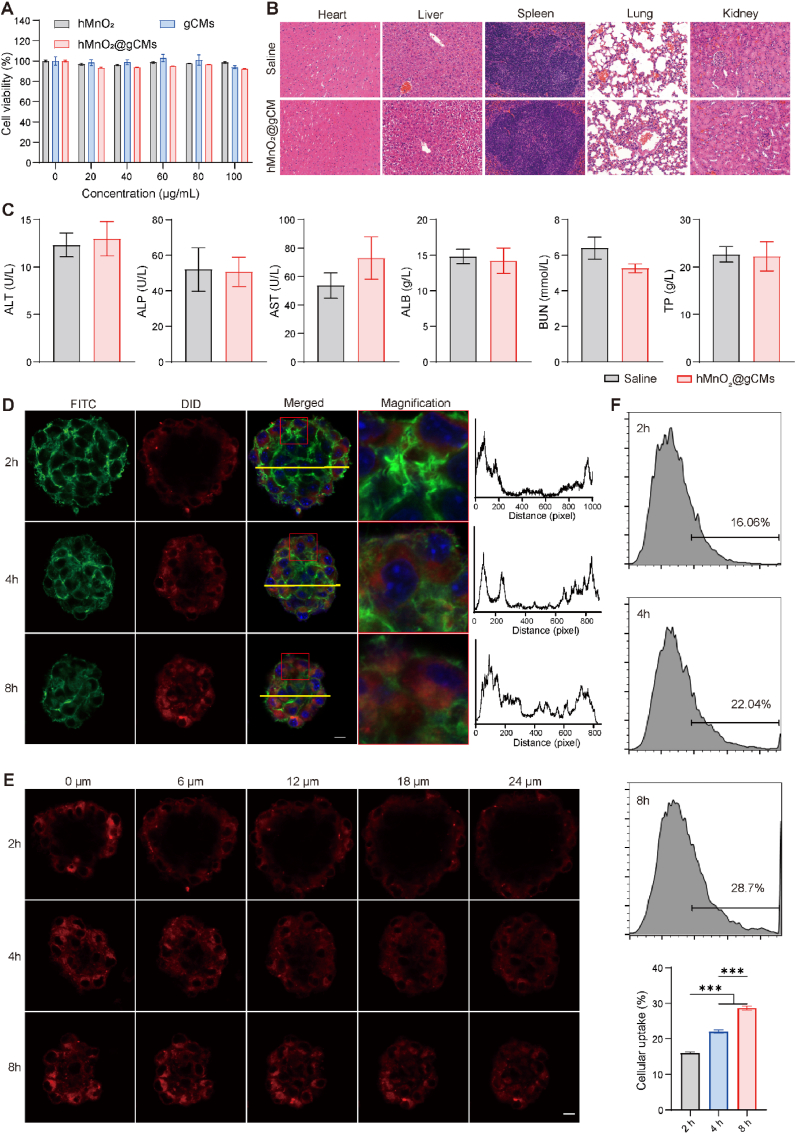
Biocompatibility and CSCs targeting ability of hMnO_2_@gCMs. (A) Viabilities of B16F10 tumorsphere cells incubated with hMnO_2_, gCMs, and hMnO_2_@gCMs. (B) H&E-stained slice images of major organs (heart, liver, spleen, lung, and kidney) of healthy C57BL/6 mice after *i.v.* injection of saline or hMnO_2_@gCMs. (C) Blood biochemistry test of hMnO_2_@gCMs. ALT: alanine transaminase, ALP: alkaline phosphatase, AST: aspartate aminotransferase, ALB: albumin, BUN: blood urea nitrogen, TP: Total protein. (D) CLSM images and the corresponding localized magnification images (red box) and line profiles (yellow lines) of B16F10 tumorsphere cells incubated with hMnO_2_@gCMs at 2 h, 4 h, 8 h. Scale bar, 10 μm. (E) CLSM images of different layers in B16F10 tumorsphere cells incubated with hMnO_2_@gCMs at 2 h, 4 h, 8 h. Scale bar, 10 μm. (F) Degree of cellular uptake of hMnO_2_@gCMs in CSCs quantified by flow cytometry with the incubation times of 2 h, 4 h and 8 h, respectively. The experimental data were presented as mean ± S.E.M. (A, *n* = 5; C, F *n* = 3). ***P < 0.001.

Firstly, the *in vitro* biocompatibility of hMnO_2_@gCMs was tested *via* the CCK8 assay [43], wherein B16F10 tumorsphere cells treated with various nanomaterials showed no obvious changes in their viability (Fig. 3A). The *in vivo* biocompatibility of hMnO_2_@gCMs was evaluated by H&E staining of major organs in healthy mice after tail vein injection. No evident tissue lesions between PBS and hMnO_2_@gCMs were observed (Fig. 3B). Moreover, there were no distinct differences in blood biochemistry (Fig. 3C) and complete blood level (Fig. S7) between PBS and hMnO_2_@gCMs at four weeks post-injection. This result indicated that the prepared hMnO_2_@gCMs had favorable biocompatibility. To verify the CSC-targeting ability of the prepared nanoparticles, we investigated the cellular uptake of hMnO_2_@gCMs in B16F10 tumorsphere cells using CLSM (Fig. S8). As the incubation time increased, stronger fluorescence of the hMnO_2_@gCMs nanocomposites was observed in the tumorsphere cells at 8 h, indicating that hMnO_2_@gCMs entered the tumorsphere cells more easily with an increase in time duration (Fig. 3D). Notably, hMnO_2_@gCMs nanocomposites were significantly observed in different layers of tumorsphere cells by changing the z-axis (Fig. 3E). Furthermore, there was a stronger fluorescence intensity in the CD133-overexpressed CSCs in B16F10 tumorsphere cells when the incubation time was increased (Fig. 3F). These results indicated that hMnO_2_@gCMs nanocomposite target CSCs through homologous targeting due to the high affinity of gCMs to CSCs. To verify lysosomal escape, CLSM was used to study the intracellular localization of hMnO_2_@gCM. The red and green signals overlapped at first and then separated over time, indicating efficient lysosomal escape of hMnO_2_@gCM (Fig. S9).

**Fig. 4:**
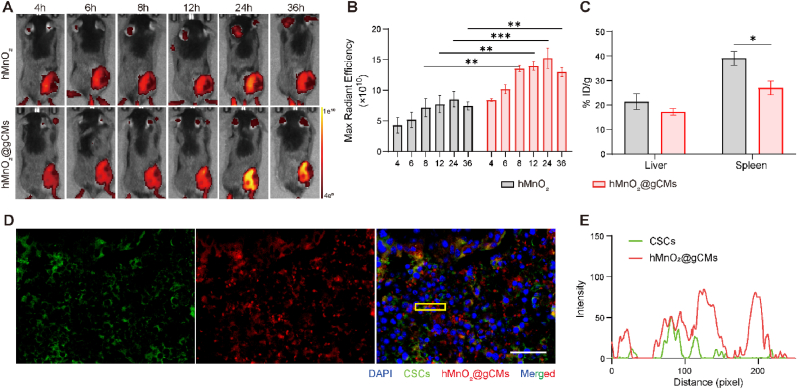
*In vivo* CSCs targeting ability of hMnO_2_@gCMs. (A) *In vivo* fluorescence imaging and (B) their quantitative analysis of B16F10–CSCs tumor-bearing mice with *i.v.* injection of hMnO_2_ and hMnO_2_@gCMs. (C) Bio-distribution of nanocomposites in different organs of B16F10–CSCs tumor-bearing mice with *i.v.* injection of hMnO_2_ and hMnO_2_@gCMs. (D, E) Immunohistochemical staining and the corresponding line profiles of hMnO_2_@gCMs (red) internalized by CSCs, CSCs were stained with CD133 (green). Scale bar, 50 μm. The experimental data were presented as mean ± S.E.M. (*n* = 3). *P < 0.05, **P < 0.01, ***P < 0.001.

Tumor targeting efficacy of hMnO_2_@gCMs was tracked by small animal imaging system, and obvious fluorescence signals appeared in the tumor area (Fig. 4A). With the gradual accumulation of nanoparticles in the tumor, the fluorescence signals gradually increased and reached the pinnacle 24 h after injection. It is encouraging to see that the fluorescence intensity was substantially higher in the case of hMnO_2_@gCMs than it was in hMnO_2_, showing that gCMs effectively improved the accumulation in the tumor (Fig. 4B). Furthermore, the Mn concentration of hMnO_2_ and hMnO_2_@gCMs in the tumor and the main organs was quantified through ICP-OES 24 h post intravenous (*i.v.*) administration (Fig. 4C and Fig. S10). Accumulation in tumors injected with hMnO_2_@gCMs was higher than hMnO_2_, while engulfment in the liver and spleen was reduced, which suggested the superior targeting ability of hMnO_2_@gCMs towards CSCs *in vivo* (Fig. 4C). The fluorescence signal of hMnO_2_@gCMs in tumors was widely detected and stronger than that of hMnO_2_ (Fig. S11). The *in vivo* targeting of hMnO_2_@gCMs to CD133-overexpressed CSCs can be confirmed by the immunofluorescent staining of the tumor sections after *i.v.* injection. As shown in Fig. 4D and E, obvious co-localization between hMnO_2_@gCMs (red) and CSCs (green) could be clearly observed. The results revealed that hMnO_2_@gCMs possessed excellent *in vivo* targeting capability to CSCs.

### 3.3 Hypoxia relief by hMnO_2_@gCMs

**Fig. 5:**
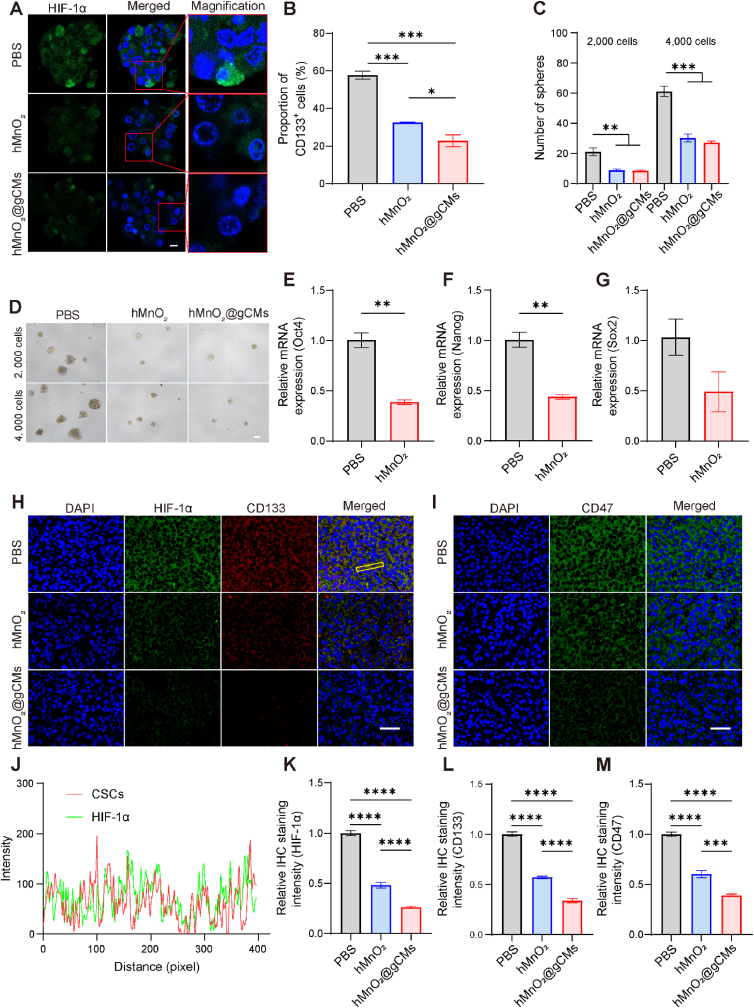
Hypoxia relief by hMnO_2_@gCMs. (A) Immunofluorescence images of HIF-1α in B16F10 tumorsphere cells after treated with PBS, hMnO_2_ and hMnO_2_@gCMs. Scale bar, 10 μm. (B) Statistic results of the percentage of CD133^+^ in B16F10 tumorsphere cells after hypoxia relief by various nanocomposites. (C) Number of spheroids (>50 μm) with 2000 or 4000 reseeded cells and (D) representative images of secondary tumorsphere growth. Scale bar, 200 μm. (E–G) Expression of stemness-associated genes (Oct4, Nanog, and Sox2) in B16F10 tumorsphere cells after various treatment. Immunofluorescence analysis of the (H) HIF-1α, CD133, and (I) CD47 of the tumor tissues after various treatment. Scale bar, 50 μm. (J) The corresponding line profiles of HIF-1α and CD133 in H. Relative Immunofluorescence intensity of the (K) HIF-1α, (L) CD133, (M) CD47 of the tumor tissues after various treatment. The experimental data were presented as mean ± S.E.M. (*n* = 3). *P < 0.05, **P < 0.01, ***P < 0.001.

High HIF-1α expression is associated with the tumor hypoxia (Fig. S12) [44]. We further investigated the expression of HIF-1α in B16F10 tumorsphere cells to evaluate the ability of hMnO_2_@gCMs to alleviate hypoxia. Under hypoxic conditions, both hMnO_2_ and hMnO_2_@gCMs treatment a conspicuous reduced the expression of HIF-1α, indicating that hypoxia was alleviated obviously (Fig. 5A). Notably, a recent study has reported that abrogation of HIF-1α expression significantly reduces the CSCs population [45,46]. To evaluate the impact of hypoxia relief on CSCs, the percentage of the CD133^+^ population, a specific marker for CSCs, was determined [47]. Fig. 5B revealed that the proportion of CD133^+^ cells decreased to 32.4 % after hMnO_2_ treatment. It is to be noted that the hMnO_2_@gCMs showed more effective reduction of CSCs, which indicated that enhanced cellular uptake could be more beneficial for hypoxia relief. Additionally, tumorspheres formation capacity was also reduced following hMnO_2_ and hMnO_2_@gCMs mediated treatment (Fig. 5C and D). Furthermore, the expression of stemness-associated genes in the tumorsphere cells decreased after the treatment (Fig. 5E to G). These results indicated that hypoxia relief has great potential to reprogram the CSC niche to reduce their stemness.

H_2_O_2_ level in cancer cells has been reported to be 50–100 μM in solid tumors [48]. The hMnO_2_ can trigger the decomposition of H_2_O_2_ and produce O_2_ *in situ* to alleviate tumor hypoxia [27]. To confirm the alleviating effect of hMnO_2_@gCMs on tumor hypoxia, tumor slices were collected 24 h after injection for HIF-1α immunofluorescence staining (Fig. 5H). Hypoxia has profound effects on the maintenance of the CSCs, hypoxia-driven CSCs enrichment is primarily mediated by HIF-1α [26]. The red signal of CSCs significantly colocalized with HIF-1α, indicating that hypoxia driven CSC enrichment (Fig. 5J). The HIF-1α signal was significantly reduced in the both hMnO_2_ and hMnO_2_@gCMs-treated group compared with the PBS group. The semi-quantitative statistical results of HIF-1α confirmed the downregulation of HIF-1α expression (Fig. 5K), indicating that the H_2_O_2_ decomposition triggered by hMnO_2_ greatly alleviated tumor hypoxia. Moreover, disrupting tumor hypoxia has been reported to effectively suppress CSCs in solid tumors [46]. The decreased expression of CD133 indicated that hMnO_2_ directly suppresses the CSCs in tumor tissues owing to the reduction of the HIF-1α (Fig. 5L).

HIF-1α increases the cell-surface expression of CD47 by directly activating the transcription of the CD47 gene in hypoxic CSCs [16]. Based on hypoxia-relieving ability, the synthesized hMnO_2_@gCMs suppressed the CD47 expression by downregulating HIF-1α expression *in vivo*. The tumor sections were extracted 24 h post injection of hMnO_2_ and hMnO_2_@gCMs for CD47 immunofluorescence staining. Compared with the PBS group, the fluorescence intensity of the hMnO_2_ treated group was significantly reduced, indicating that the downregulation of HIF-1α expression by hypoxia relief was able to inhibit CD47 expression (Fig. 5I). In addition, due to the CSC targeting effect, the hMnO_2_@gCMs treatment group showed better results in reducing CD47 expression (Fig. 5M). Overexpressed CD47 can interact with SIRPα on the surface of macrophages to induce immune evasion by blocking phagocytosis [38,39]. Given that the binding between SIRPα variant and CD47 can trigger the macrophage phagocytosis of the cancer cells, the hypoxia relief-induced downregulation of CD47 and immune checkpoints blockade therapy could synergistically inhibit the CD47-SIRPα signaling pathway, thereby eliciting a significant immune response.

### 3.4 Antitumor immune activation by hMnO_2_@gCMs

**Fig. 6:**
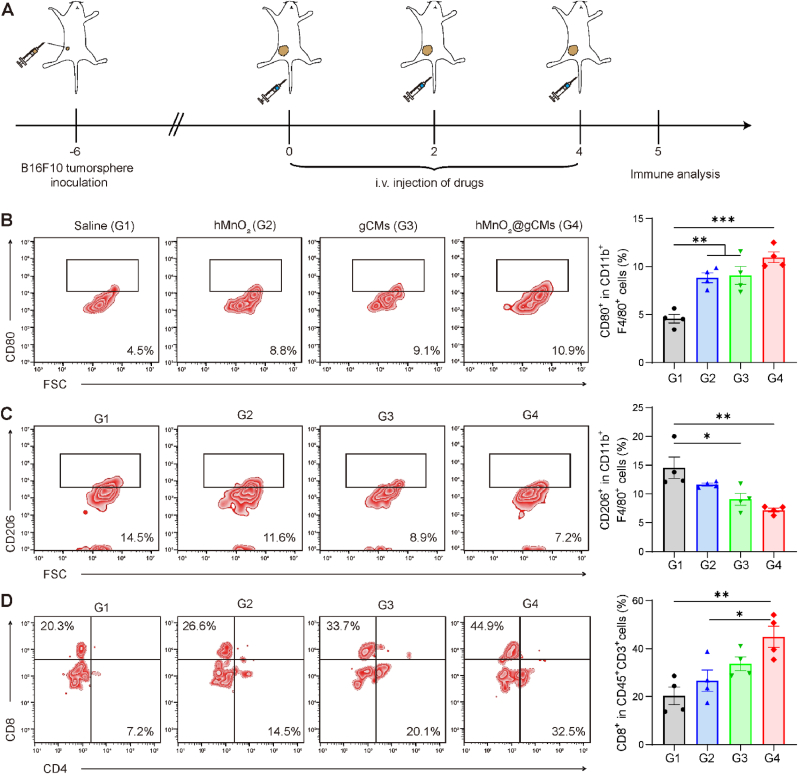
Antitumor immune activation by hMnO_2_@gCMs. (A) Establishment of subcutaneous B16F10–CSCs tumor bearing mice model and schematic diagram of treatment timeline. FACS analysis of (B) M1-like macrophages (CD80^+^) and (C) M2-like macrophages (CD206^+^) in tumor tissues. (D) FACS analysis of CD8^+^ T cells in tumor tissues. The experimental data were presented as mean ± S.E.M. (*n* = 4). *P < 0.05, **P < 0.01, ***P < 0.001.

To study the immune status of tumor tissues after treatment, immune cell in the tumor site were analyzed by FACS (Fig. 6A), and the gating strategy of the cells was shown in Fig. S13. A hypoxic tumor microenvironment (TME) has been reported to impede effective tumor immunotherapy, which could tune the TAM into M2-like phenotype cells, thereby promoting tumor progression by suppressing anti-tumor immunities [27]. To investigate whether alleviating hypoxia in tumors can promote immunotherapy efficacy, the proportion of macrophages in the tumor tissues were analyzed by FACS after treatments. Given the ability of hMnO_2_ to alleviate tumor hypoxia, we investigated whether the given treatment had an impact on the tumor immunology. Compared with the untreated group, the infiltration of M1 phenotype TAMs was significantly increased in hMnO_2_-treated tumors (Fig. 6B), while the percentage of M2 phenotype TAMs was greatly reduced (Fig. 6C). M2 phenotype TAMs are associated with immunosuppression, tumors treated with hMnO_2_ showed higher M1 to M2 ratios (Fig. S14), indicating the hypoxia relief could improve the immunosuppressive TME. Moreover, blocking the CD47-SIRPα could trigger macrophage-mediated cancer cell phagocytosis and promote antitumor immune responses [38], the gCM treatment increased M1 phenotype TAMs and reduced M2 phenotype TAMs in tumor tissues. The proportion of M1 phenotype TAMs in hMnO_2_@gCMs-treated group was higher than that in hMnO_2_ and gCM -treated group, indicating that that the synergistic effect of hypoxia relief-induced downregulation of CD47 and immune checkpoints blockade therapy greatly enhanced the immune response.

**Fig. 7:**
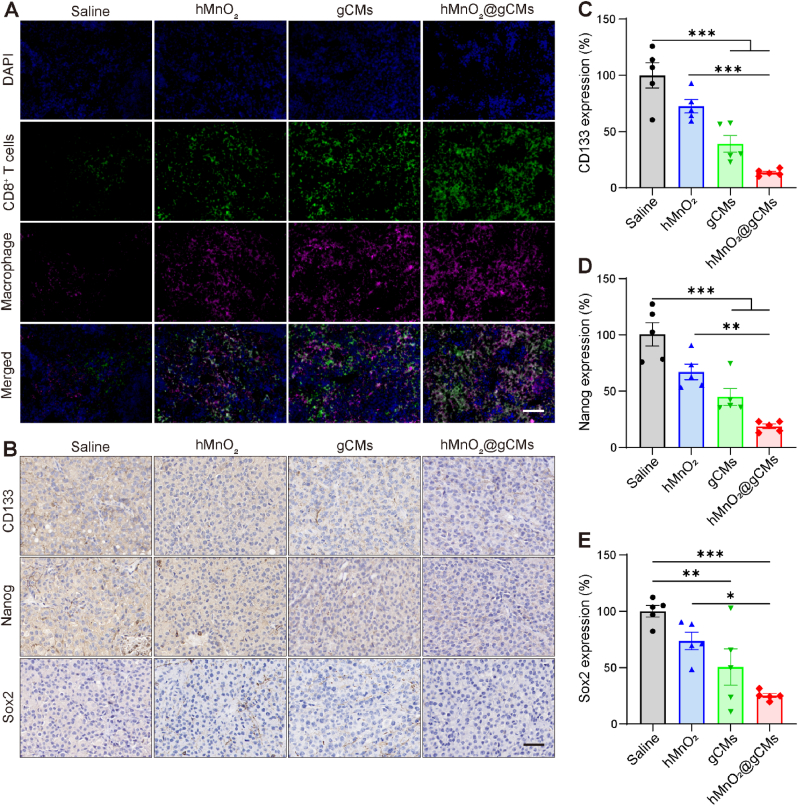
Antitumor immune activation and CSC elimination by hMnO_2_@gCMs. (A) Immunofluorescence analysis of CD8^+^ T cell and macrophage infiltration in tumors tissues after receiving different treatments. Scale bar, 50 μm. (B) Immunohistochemical staining of the tumor CD133, Nanog, and Sox2 of B16F10–CSCs tumor-bearing mice in each group at the end of therapy. Scale bar, 50 μm. Relative immunohistochemical staining intensity of the (C) CD133, (D) Nanog, (E) Sox2 in B16F10–CSCs tumor-bearing mice in each group at the end of therapy. The experimental data were presented as mean ± S.E.M. (*n* = 5). *P < 0.05, **P < 0.01, ***P < 0.001.

It was found that compared with the untreated group, there were more CD8^+^ cytotoxic T cells and CD4^+^ helper T cells infiltrated in the tumor after hMnO_2_ and gCMs treatment (Fig. 6D and Fig. S15), implying that the treatment could effectively stimulate antitumor immune responses. In addition, tumors treated with hMnO_2_@gCMs had the maximal infiltration of CD8^+^ T cells and CD4^+^ helper T cells compared with other groups. Similarly, the immunofluorescence staining observations showed that combined hypoxia relief and synergetic inhibition of CD47-SIRPα treatment with hMnO_2_@gCMs exhibited a higher level of macrophages and CD8^+^ T cells infiltration in the tumor tissue (Fig. 7A). Considering hypoxia relief-induced CD47 downregulation and the inhibition of CD47-SIRPα axis, CSCs could more easily be eliminated by the infiltrating M1 macrophages and CD8^+^ T cells. Furthermore, compared with the other groups, increased expression of IFN-γ and TNF-α was found in mice injected with hMnO_2_@gCMs, further confirming that hypoxia alleviation combined with synergetic inhibition of CD47-SIRPα exhibited robust anti-tumor immunity (Fig. S16).

### 3.5 CSC elimination and tumor growth inhibition by hMnO_2_@gCMs

CSCs have been reported to affect tumor formation and growth, thereby reducing therapeutic efficacy [49,50]. Next, we investigated the effect of various treatment on CSCs by utilizing CSCs markers including CD133, Nanog and Sox2 (Fig. 7B). The hMnO_2_ and gCMs treated group reduced the proportion of CSCs, notably, hMnO_2_@gCMs treated group significantly promoted the elimination of CSCs compared with other groups. The enhanced suppression of CSCs was attributed to CSC targeting and hypoxia relief combined with synergetic inhibition of the CD47-SIRPα. In addition, the corresponding relative IHC staining intensities were consistent with the results of IHC staining images (Fig. 7C–E). These results demonstrated the excellent ability of hMnO_2_@gCMs to effectively target and eliminate CSCs *in vivo.*

**Fig. 8:**
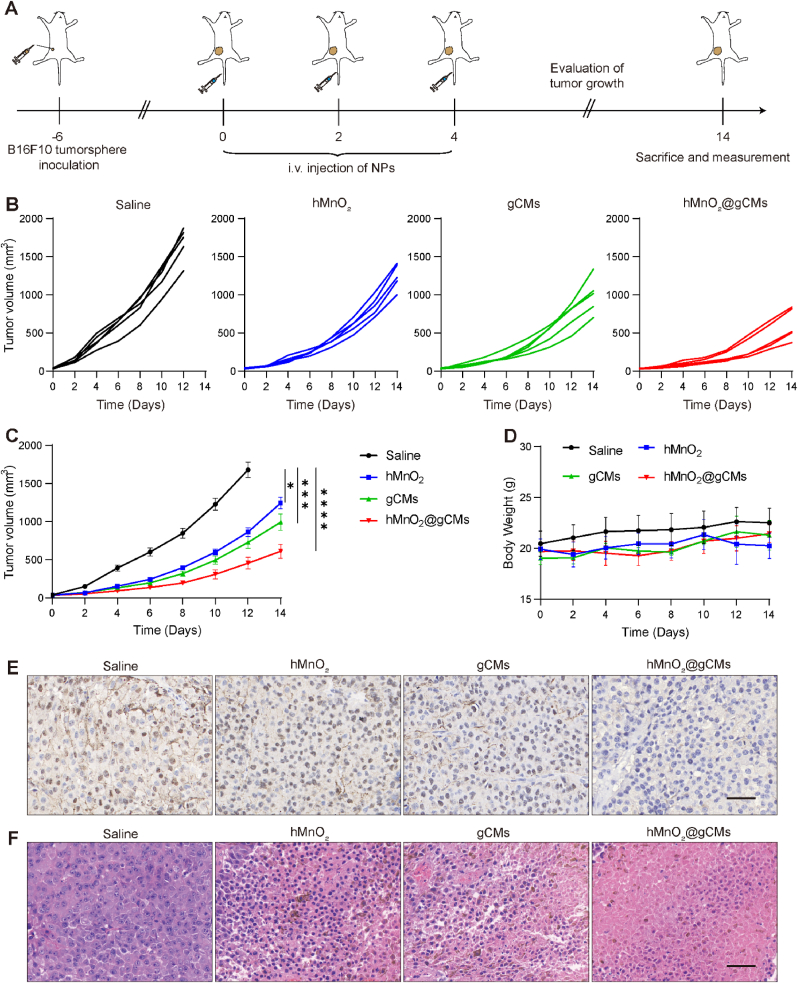
Tumor growth inhibition by hMnO_2_@gCMs. (A) Establishment of subcutaneous B16F10–CSCs tumor bearing mice model and schematic diagram of treatment timeline. (B) Individual, (C) average tumor growth profiles and (D) body weight change of the B16F10–CSCs tumor-bearing mice during the two-week therapy. (E) Immunohistochemical staining of the tumor Ki-67 of B16F10–CSCs tumor-bearing mice in each group at the end of therapy. Scale bar, 50 μm. (F) H&E staining of B16F10–CSCs tumor-bearing mice in each group at the end of therapy. Scale bar, 50 μm. The experimental data were presented as mean ± S.E.M. (*n* = 5). *P < 0.05, ***P < 0.001, ****P < 0.0001.

Subsequently, we investigated the *in vivo* therapeutic effect of hMnO_2_@gCMs on CSCs (Fig. 8A). The relative tumor volume of hMnO_2_ treated group was smaller than that of the PBS group (Fig. 8B–C). Mice in the gCMs treated group showed higher tumor suppression, implying that blocking CD47-SIRPα “don't eat me” signaling pathway is crucial for the elimination of CSCs. Furthermore, tumors treated with hMnO_2_@gCMs exhibited significant growth inhibition compared with the hMnO_2_ and gCMs treated groups, indicating that the hypoxia relief combined with synergetic inhibition of the CD47-SIRPα improved the *in vivo* therapeutic efficiency. The hMnO_2_@gCMs treated group exhibited least Ki-67 labeled cell proliferation (Fig. 8E), indicating that hMnO_2_@gCMs treatment successfully inhibited the CSCs proliferation. Moreover, prominently dark colors could be observed in H&E staining of the three treated tumor tissue (Fig. 8F). Additionally, during the two weeks of treatment, no significant weight fluctuations was observed in any group (Fig. 8D), no obvious tissue damage, necrosis and fibrosis was observed in the H&E-staining of organ tissue section (Fig. S17), indicating the hMnO_2_@gCMs nanoparticles did not induce adverse side effects during the treatments. The *in vivo* therapeutic efficacy results suggested that hMnO_2_@gCMs achieved excellent anti-tumor efficiency.

Conventional chemotherapy cannot effectively kill CSCs, and the combination of immunotherapy and chemotherapy has been recognized as an effective treatment strategy to improve the therapeutic efficacy of CSCs [5]. To investigate the *in vivo* therapeutic efficiency of combined chemo-immunotherapy on CSCs, chemotherapy drug doxorubicin (DOX) was loaded into hMnO_2_@gCMs nanoplatform, which was denoted as hMnO_2_@gCMs (DOX) (Fig. S18A). Compared with the control group, DOX treatment slightly reduced tumor growth, while hMnO_2_@gCMs treatment had significant tumor inhibition efficiency, and the hMnO_2_@gCMs (DOX) has the best tumor suppressive efficacy, indicating that the efficacy is enhanced after the combination of immunotherapy and chemotherapy (Figs. S18B and C). There was no significant change in the body weight of tumor-bearing mice after different treatments, indicating that all treatments showed good biosafety profiles (Fig. S18D).

## 4 Conclusion

In summary, we have successfully developed a genetically engineered CSC membrane-coated hMnO_2_@gCMs biomimetic nanoplatform, which exhibited an excellent hypoxia relief and immune checkpoint blockade ability. The hMnO_2_@gCMs biomimetic nanoplatform exhibited CSC-specific targeted ability through the homologous targeting ability of the CSC membrane. The obtained hMnO_2_ could generate O_2_ to relieve tumor hypoxia, thereby suppressing CSCs and reducing the expression of CD47 to enhance immune response. Moreover, the overexpressed SIRPα on gCMs enhanced the cancer immunotherapy by disrupting the CD47-SIRPα signaling pathway. The downregulation of CD47 and immune checkpoints blockade therapy performed a synergetic inhibition of the CD47-SIRPα signaling pathway, which elicited a significant immune response. In B16F10–CSCs bearing mice melanoma model, tumor hypoxia relief combined with synergetic inhibition of the CD47-SIRPα signaling pathway presented a robust effect in eradicating CSCs and inhibiting tumor growth. Therefore, this biomimetic nanoplatform showed tremendous promise for eliminating CSCs, which is encouraging for clinical applications.

## Ethics approval and consent to participate

All the animal experiments were performed in accordance with the guidelines approved by the animal research ethics committee at Shenzhen Bay Laboratory (Permit No. AERL202203).

## CRediT authorship contribution statement

**Yuanwei Pan:** Writing – review & editing, Writing – original draft, Methodology, Investigation, Conceptualization. **Ling Yu:** Conceptualization, Investigation, Methodology, Supervision, Validation. **Lujie Liu:** Writing – original draft, Validation, Methodology, Investigation. **Jing Zhang:** Writing – original draft, Validation, Methodology, Investigation. **Shuang Liang:** Writing – original draft, Validation, Methodology. **Badri Parshad:** Methodology, Investigation. **Jialin Lai:** Validation, Methodology. **Li-Min Ma:** Writing – review & editing, Validation, Supervision. **Zhaohui Wang:** Writing – review & editing, Supervision, Funding acquisition. **Lang Rao:** Writing – review & editing, Writing – original draft, Supervision, Funding acquisition, Conceptualization.

## Declaration of competing interest

The authors declare no conflict of interest in this work.
