# Supplementary material for: Genetically engineered nanomodulators elicit potent immunity against cancer stem cells by checkpoint blockade and hypoxia relief
Source: Bioact Mater. 2024 Apr 23;38:31–44. doi: 10.1016/j.bioactmat.2024.04.008 (PMC11061653; doi:10.1016/j.bioactmat.2024.04.008)
Supplement: Multimedia component 1 [file mmc1.docx]

*Supporting Information*

**Genetically Engineered Nanomodulators Elicit Potent Immunity against Cancer Stem Cells by Checkpoint Blockade and Hypoxia Relief**

Pan et al.


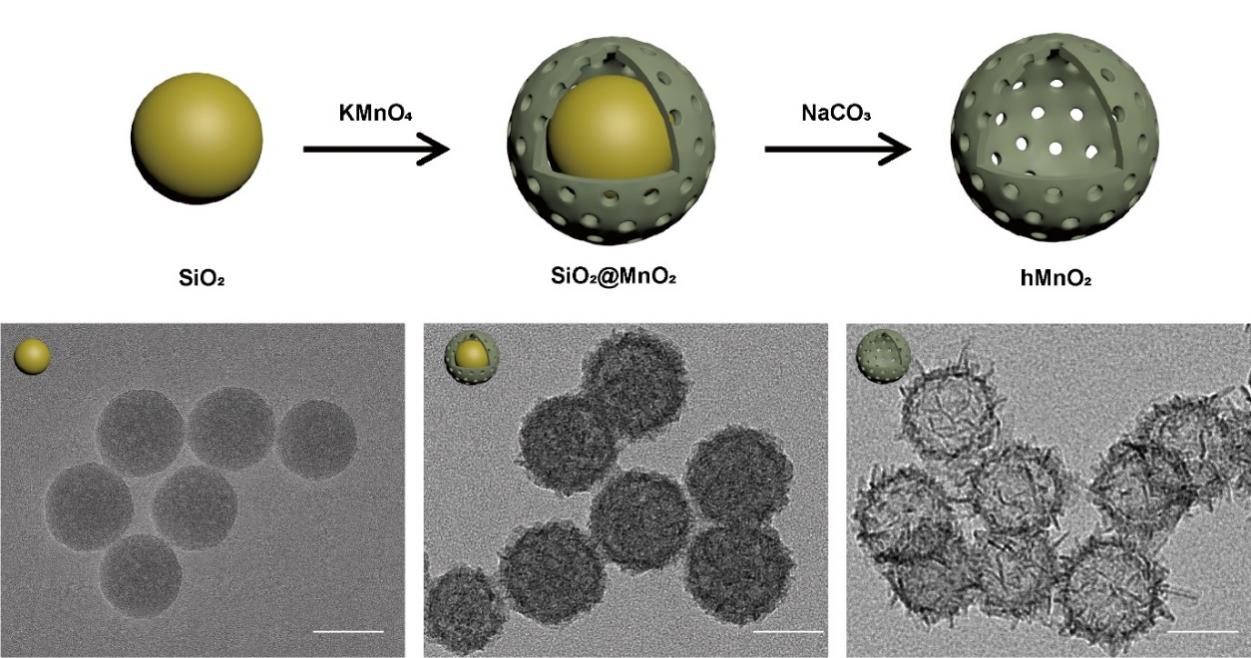


**Figure S1.** Schematic diagram of the synthesis process of hMnO_2_ nanoparticles and TEM images of nanoparticles corresponding to each step. Scale bar, 50 nm.


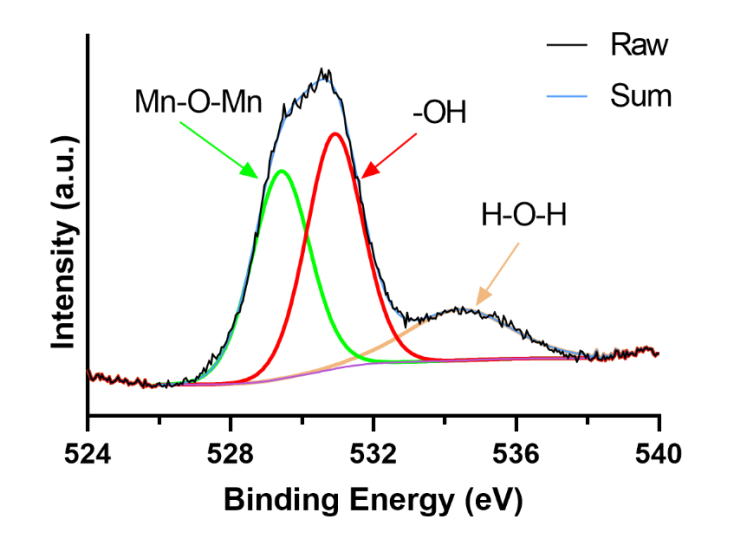


**Figure S2.** XPS spectrum of O1s of hMnO_2_ nanoparticles.


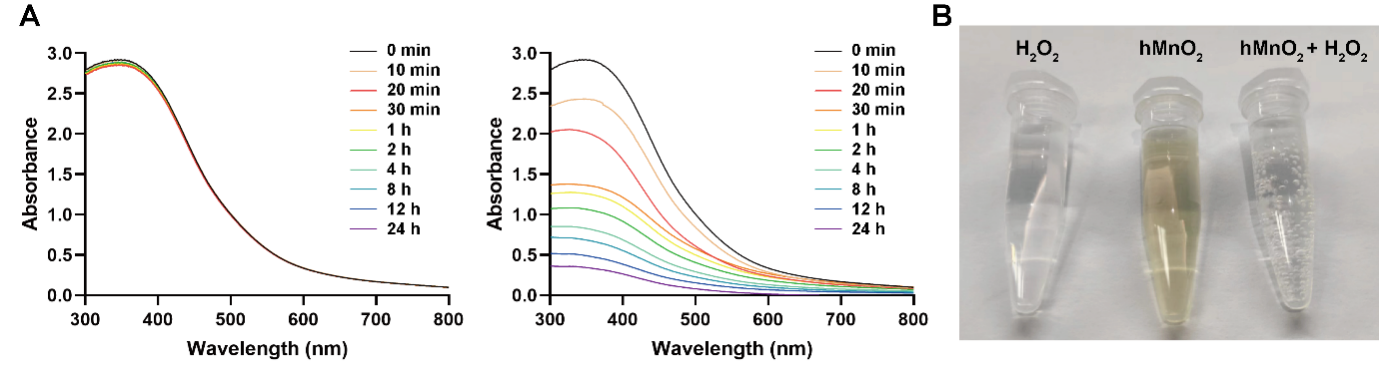


**Figure S3.** (A) The degradtion behavior of hMnO_2_ incubated in PBS (left) and H_2_O_2_ (right) measured by UV-vis spectra. (B) The photograph changes of H_2_O_2_ solutions, hMnO_2_ solutions and hMnO_2_ solutions added with H_2_O_2_.


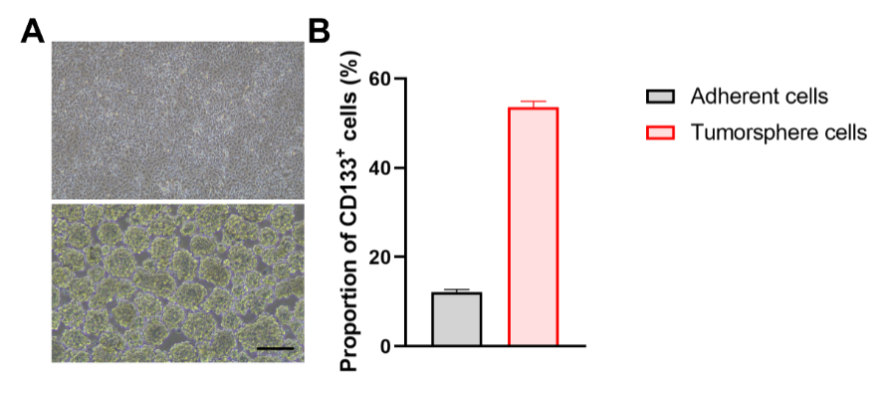


**Figure S4.** (A) Optical micrographs of B16F10-SIRPα adherent cells and tumorsphere cells. Scale bar, 100 μm. (B) Statistic results of the percentage of CD133^+^ in B16F10-SIRPα adherent cells and tumorsphere cells. The experimental data were presented as mean ± S.E.M. (*n* = 3).


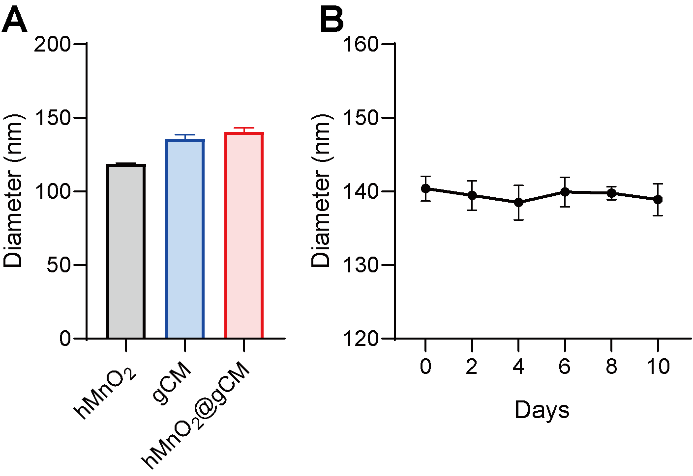


**Figure S5.** (A) The hydrated particle size of hMnO_2_, gCMs and hMnO_2_@gCMs. (B) The size changes of hMnO_2_@gCMs over a 10 days observation period. The experimental data were presented as mean ± S.E.M. (*n* = 3).


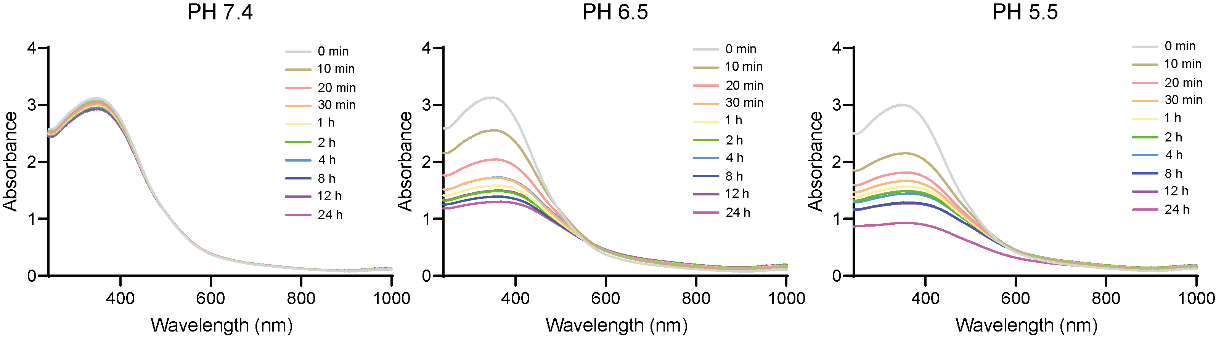


**Figure S6.** The degradation behavior of hMnO_2_@gCM incubated in PBS at different pH values (7.4, 6.5 and 5.5) measured by UV-vis spectra.


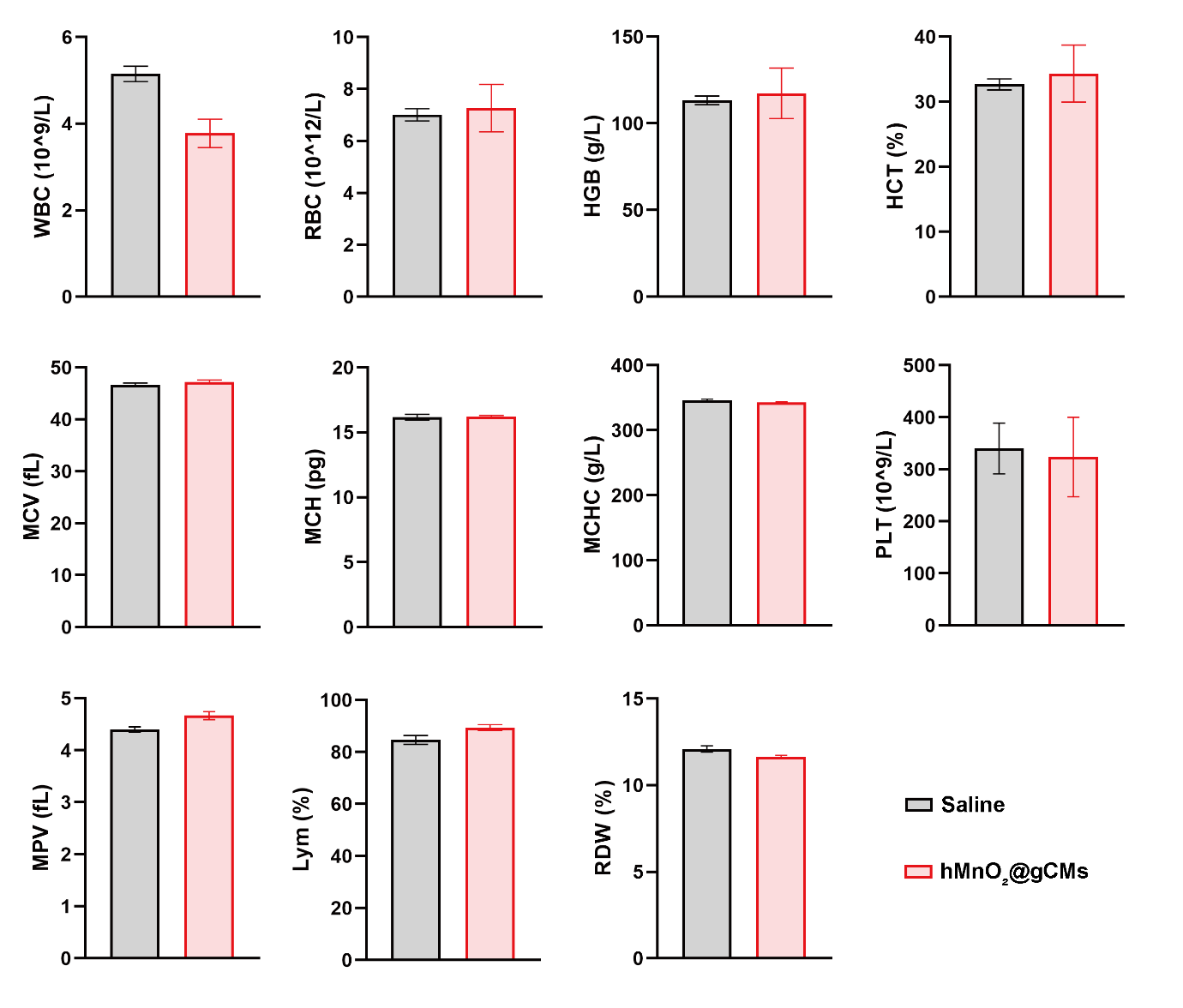


**Figure S7.** Complete blood test of healthy C57BL/6 mice after *i.v.* injection of saline or hMnO_2_@gCMs. WBC: white blood cell, RBC: red blood cell, HGB: hemoglobin, HCT: hematocrit, MCV: mean corpuscular volume, MCH: mean corpuscular hemoglobin, MCHC: mean corpuscular hemoglobin concentration, PLT: platelets, MPV: mean platelet volume, Lym: lymphocyte, RDW: red blood cell distribution width. The experimental data were presented as mean ± S.E.M. (*n* = 3).


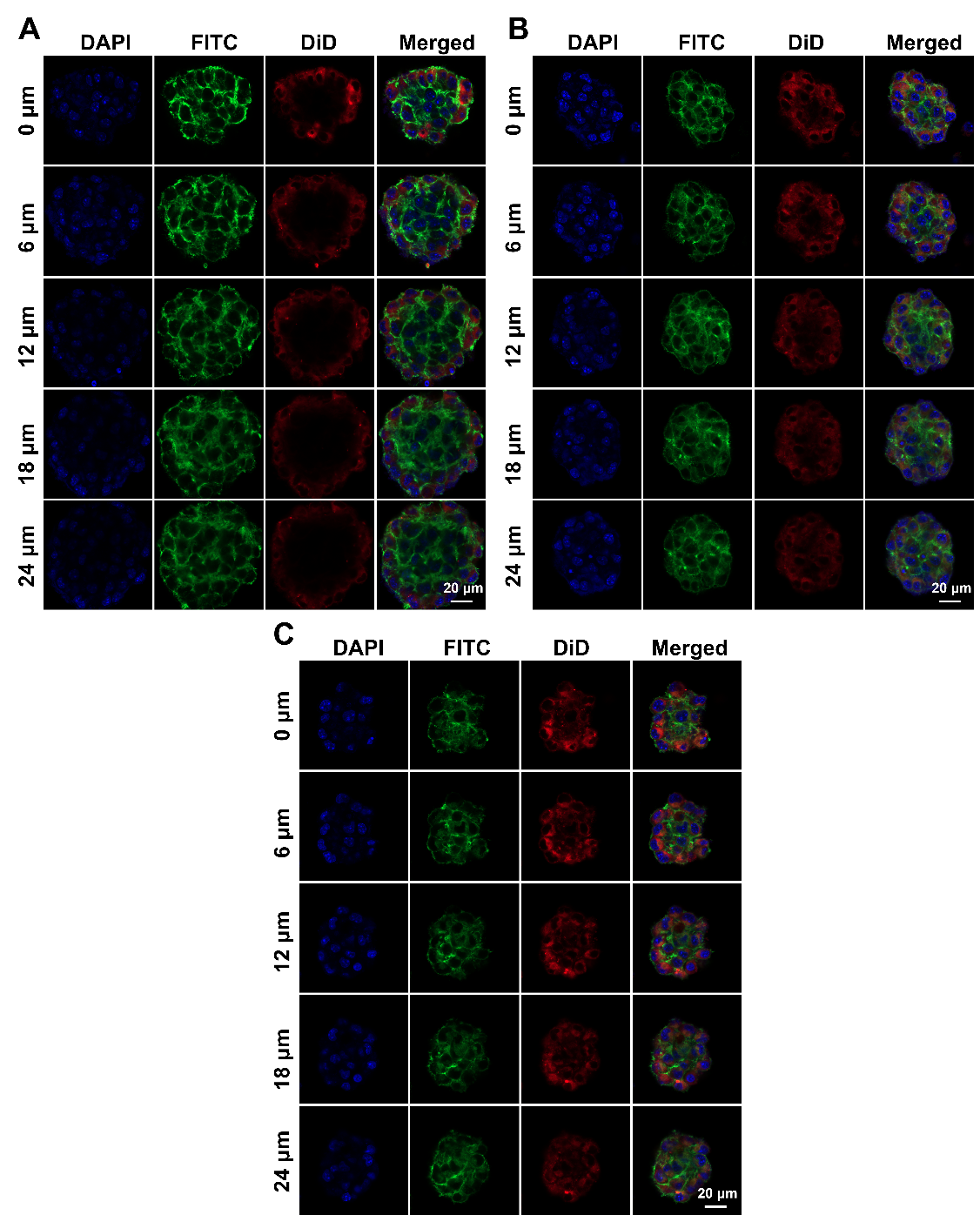


**Figure S8.** CLSM images of B16F10 tumorsphere cells incubated with hMnO_2_@gCMs at (A) 2h, (B) 4h, and (C) 8h.


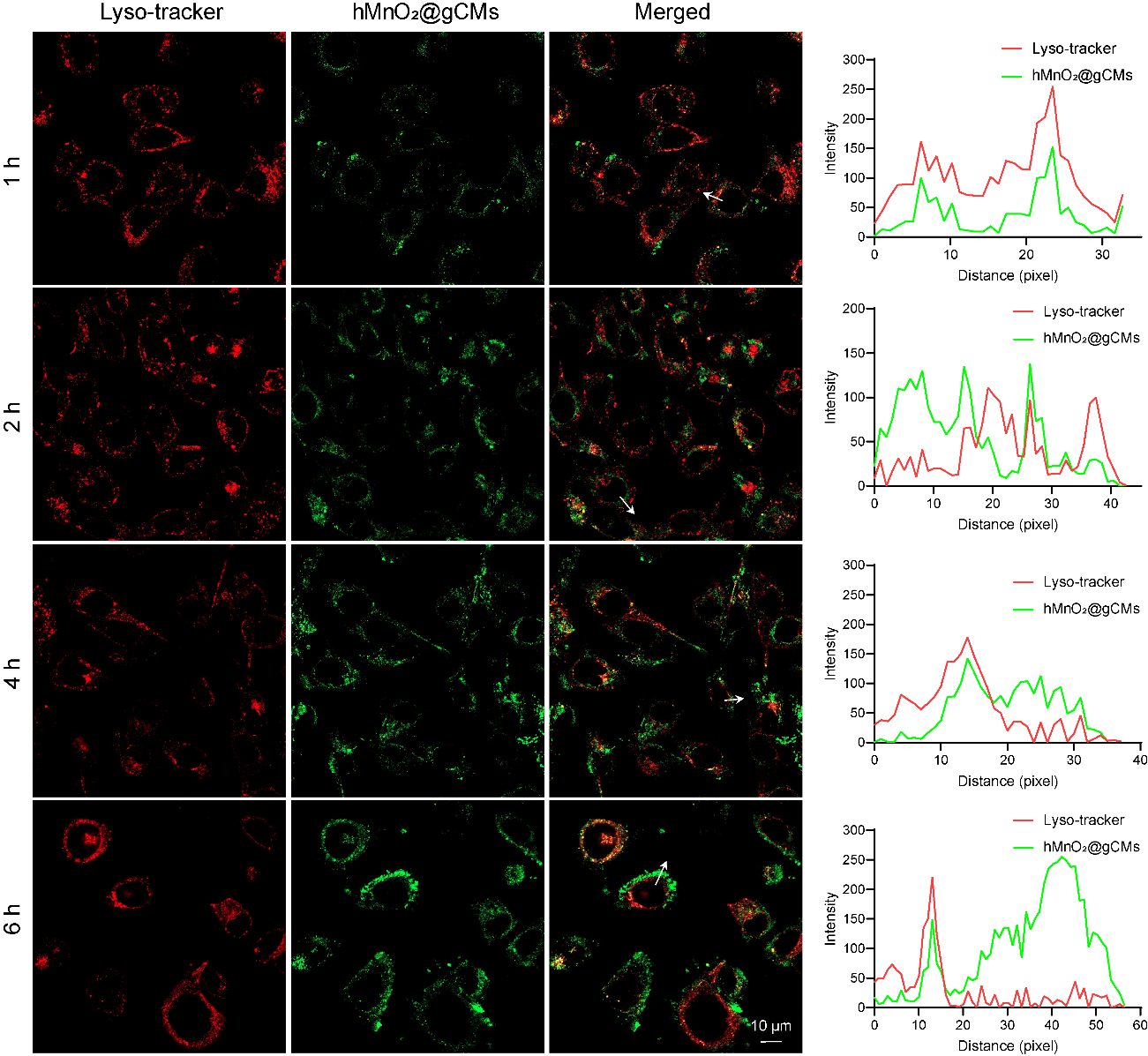


**Figure S9.** CLSM images and the corresponding line profiles of CSCs treated with DIO-labeled hMnO_2_@gCMs for different time.


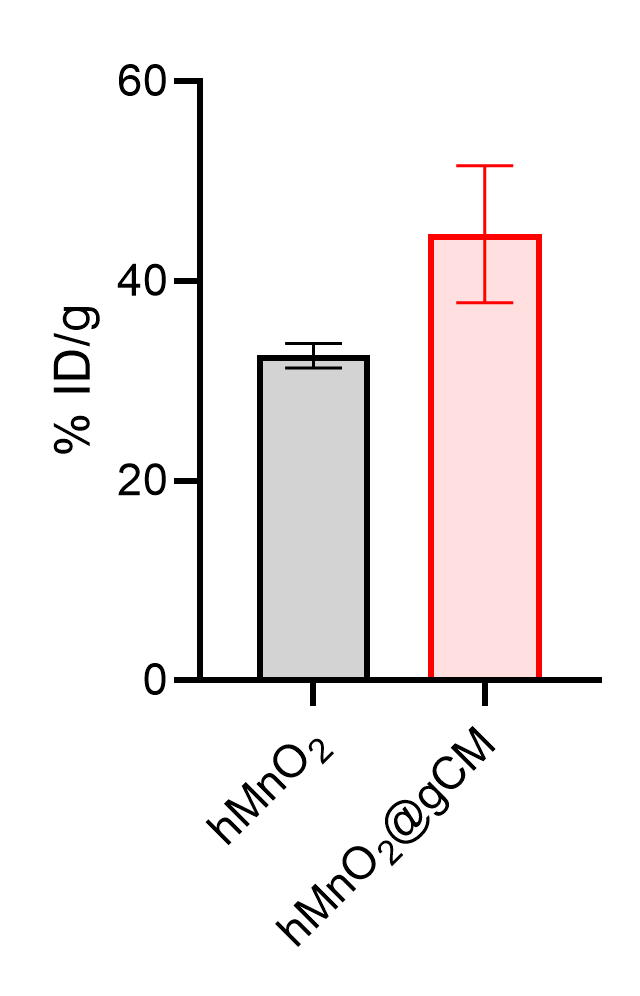


**Figure S10.** Bio-distribution of nanocomposites in tumor of B16F10-CSCs tumor-bearing mice with *i.v.* injection of hMnO_2_ and hMnO_2_@gCMs quantified by ICP-OES. The experimental data were presented as mean ± S.E.M. (*n* = 3).


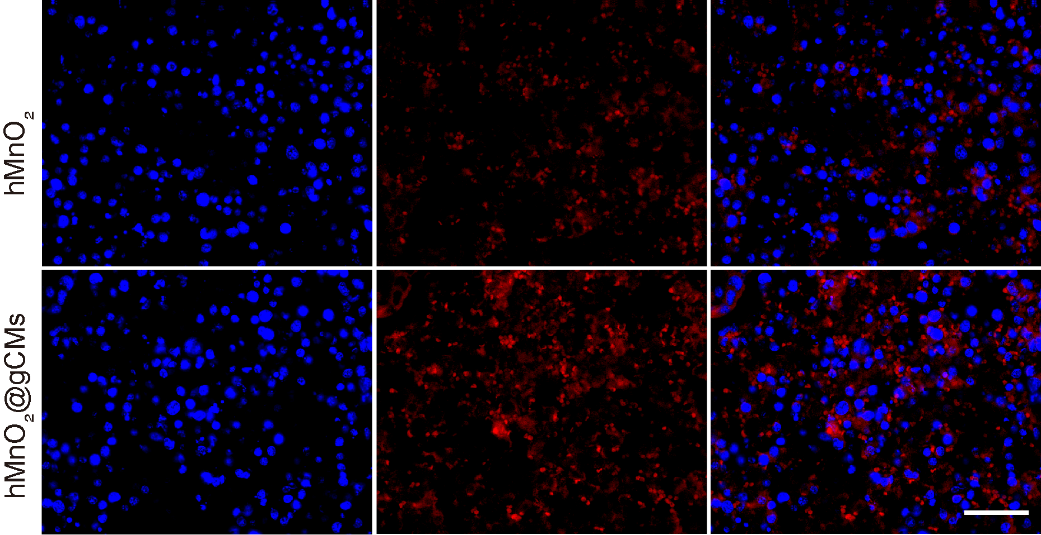


**Figure S11.** The immunofluorescent analysis of hMnO_2_ and hMnO_2_@gCMs in tumor of B16F10-CSCs tumor-bearing mice. Scale bar, 50 μm.


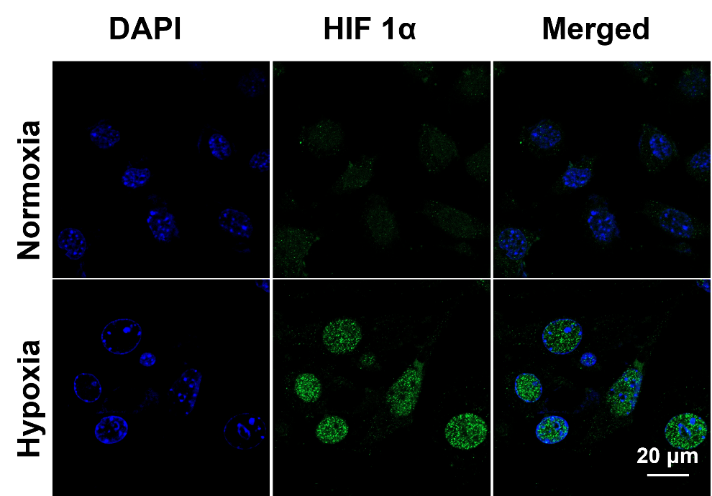


**Figure S12.** Immunofluorescence images of HIF-1α in normoxic and hypoxic environments, respectively.


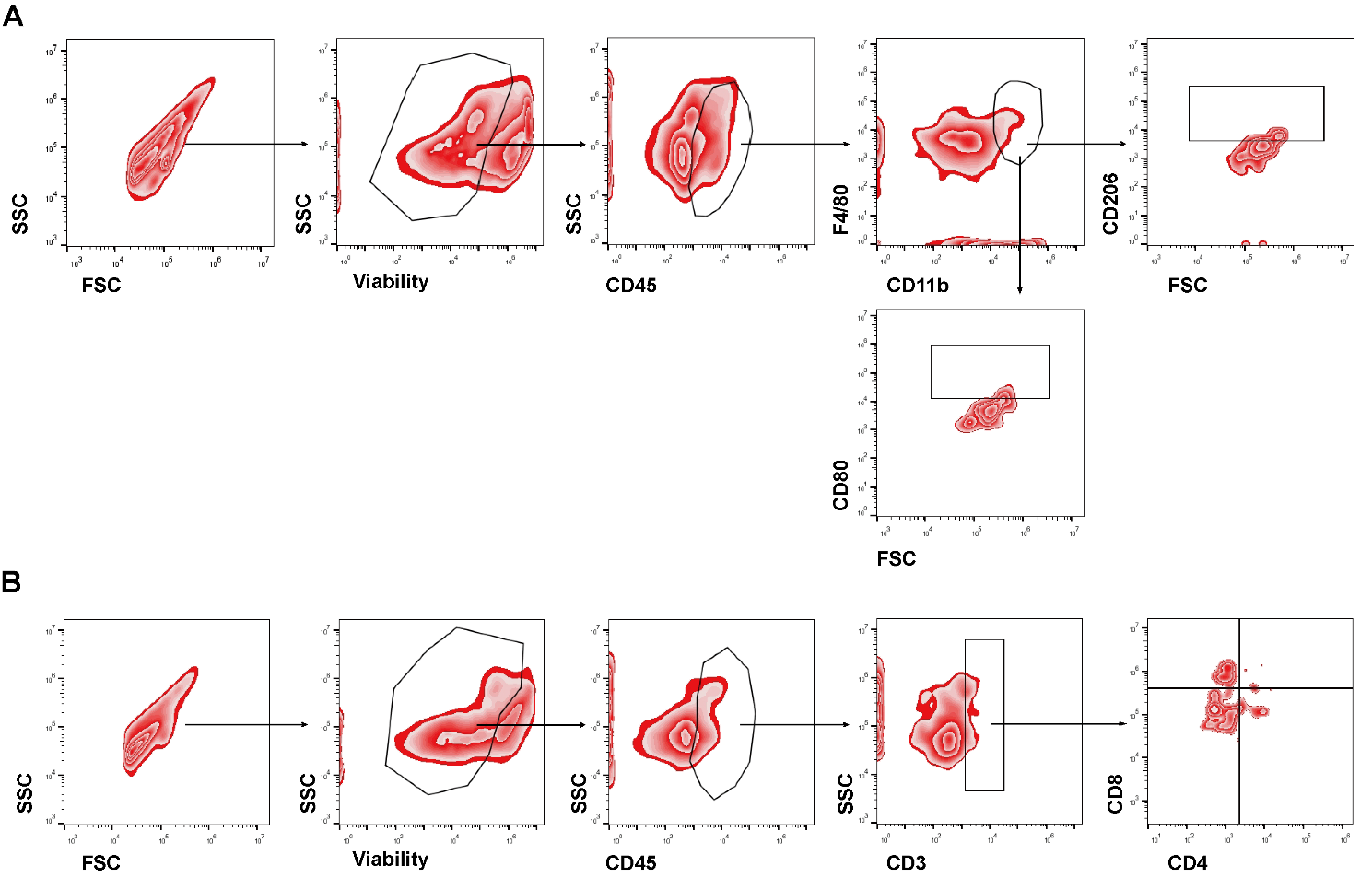


**Figure S13.** (A) Gating strategy to sort CD206^+^ M2-like and CD80^+^ M1-like macrophages in tumor tissues gating on F4/80^+^CD11b^+^CD45^+^ cells presented on Fig. 6B and 6C. (B) Gating strategy to sort CD8^+^ and CD4^+^ T cells in tumor tissues gating on CD45^+^ cells presented on Fig. 6D.


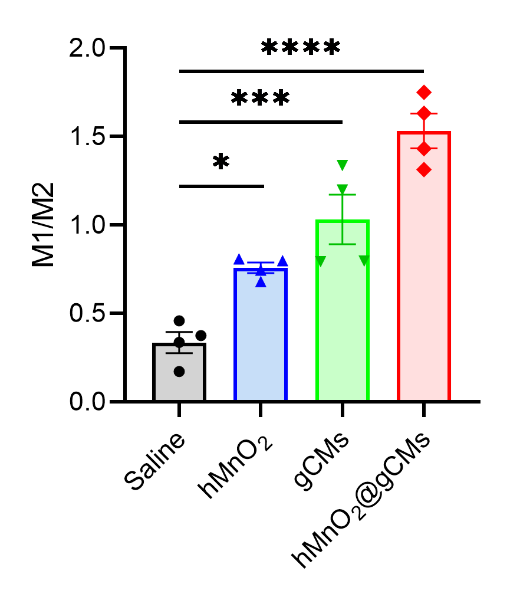


**Figure S14.** FACS analysis of M1/M2 macrophages in tumor tissues. The experimental data were presented as mean ± S.E.M. (*n* = 4). **P* < 0.05, ***P* < 0.01, ****P* < 0.001, *****P* < 0.0001.


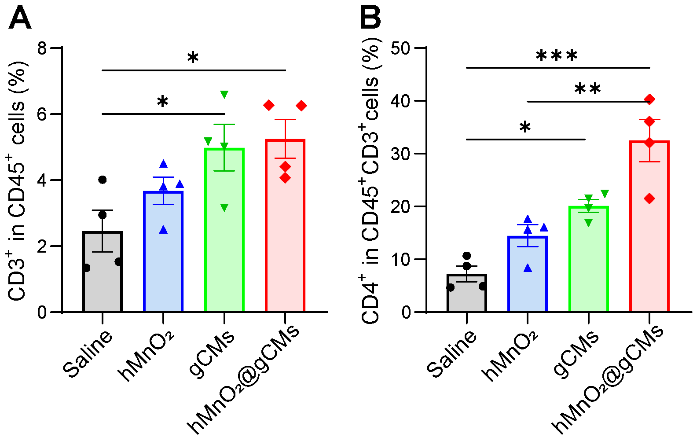


**Figure S15.** FACS analysis of (A) CD3^+^ and (B) CD4^+^ T cells in tumor tissues. The experimental data were presented as mean ± S.E.M. (*n* = 4). **P* < 0.05, ***P* < 0.01, ****P* < 0.001.


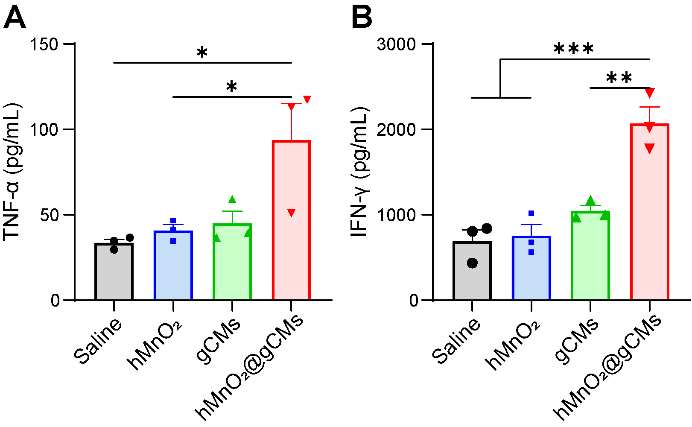


**Figure S16.** (A) TNF-α and (B) IFN-γ levels in tumor tissues collected from mice after different treatments. The experimental data were presented as mean ± SD. (*n* = 3). **P* < 0.05, ***P* < 0.01, ****P* < 0.001.


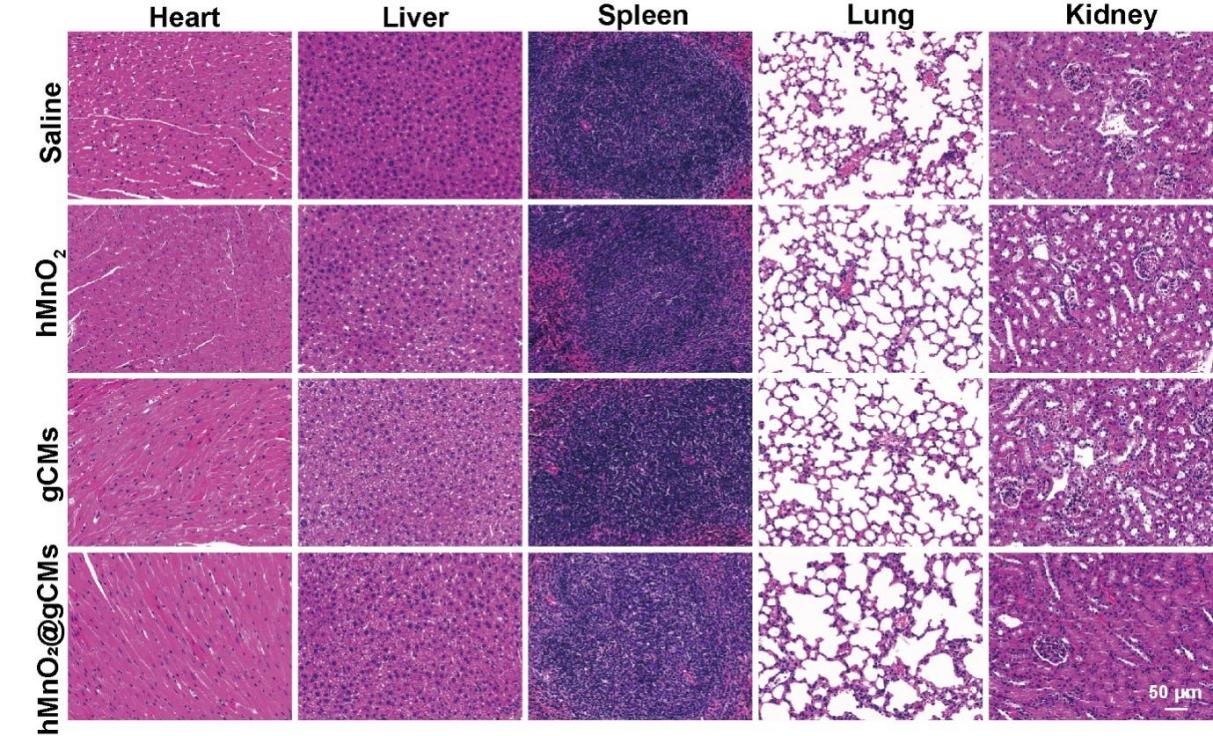


**Figure S17.** H&E staining images of major organs (heart, liver, spleen, lung, and kidney) of tumor-bearing mice in each group at the end of therapy.


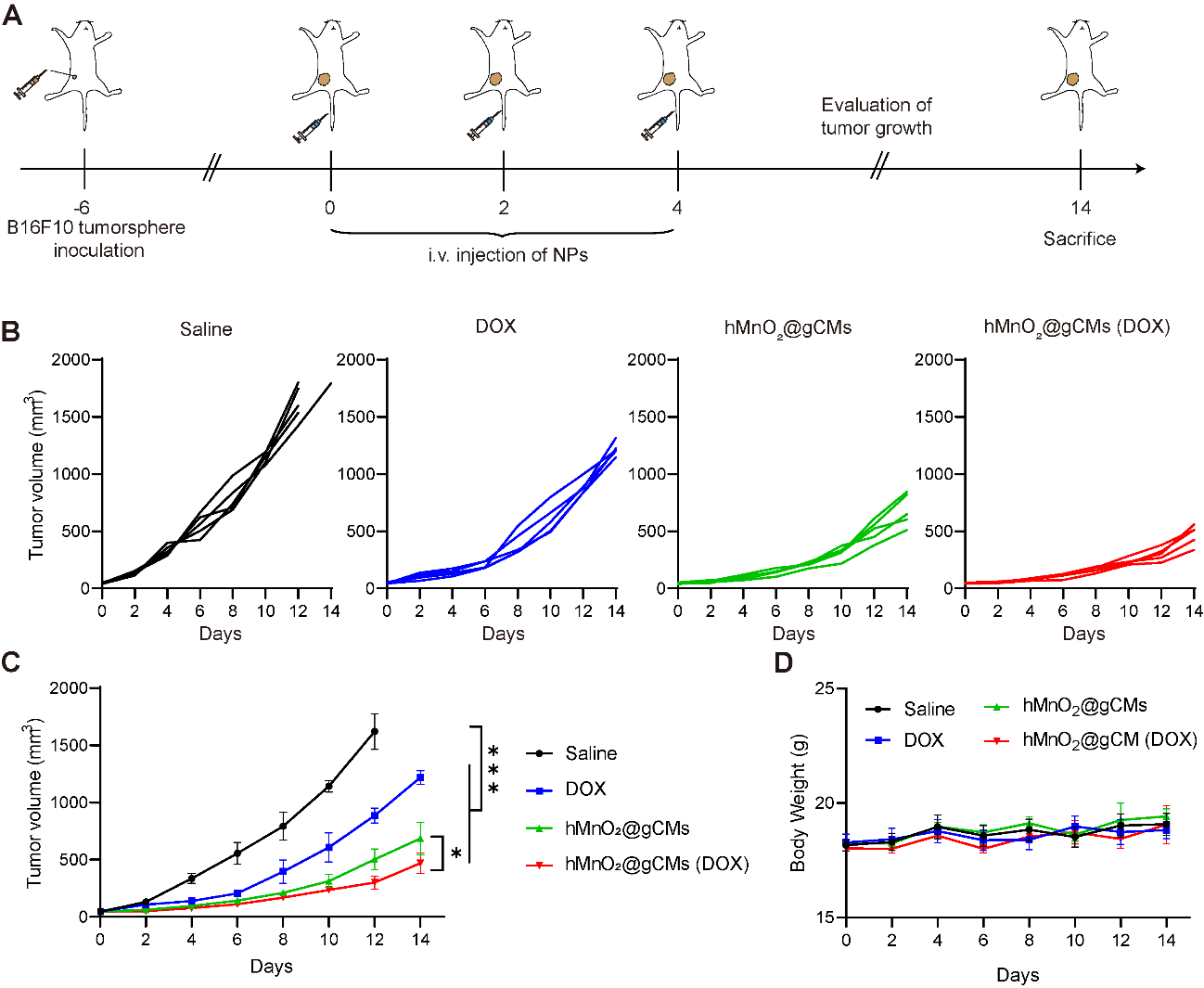


**Figure S18.** (A) Schematic diagram of treatment timeline. (B) Individual, (C) average tumor growth profiles and (D) body weight changes of the B16F10-CSCs tumor-bearing mice during a two-week treatment period. The experimental data were presented as mean ± S.E.M. (*n* = 5). **P* < 0.05, ****P* < 0.001.

**Table S1.** Primer sequences of target genes for qRT-PCR.

| Gene | Forward Primers (5’-3’) | Reverse Primers (5’-3’) |
| --- | --- | --- |
| GAPDH | ATCAAGAAGGTGGTGAAGCAGGCA | TGGAAGAGTGGGAGTTGCTGTTGA |
| Oct4 | TGGCGTGGAGACTTTGCA | GAGGTTCCCTCTGAGTTGCTTTC |
| Nanog | TCTTCCTGGTCCCCACAGTTT | GCAAGAATAGTTCTCGGGATGAA |
| Sox2 | GCACATGAACGGCTGGAGCAACG | TGCTGCGAGTAGGACATGCTGTAGG |
